# Mindfulness Meditation vs Escitalopram for Treatment of Anxiety Disorders: Secondary Analysis of a Randomized Clinical Trial

**DOI:** 10.1001/jamanetworkopen.2024.38453

**Published:** 2024-10-09

**Authors:** Hiroe Hu, Mihriye Mete, Neil K. Rustgi, Charisma I. Washington, Kavya Sanghavi, Mary Ann Dutton, Naomi M. Simon, Amanda W. Baker, Eric Bui, Elizabeth A. Hoge

**Affiliations:** 1 Experimental Therapeutics and Pathophysiology Branch, National Institute of Mental Health, National Institutes of Health, Bethesda, Maryland; 2 Medstar Health Research Institute, Hyattsville, Maryland; 3 Department of Psychiatry, Georgetown University Medical Center, Washington, DC; 4 Department of Psychiatry, NYU Grossman School of Medicine, New York; 5 Department of Psychiatry, Massachusetts General Hospital, Harvard Medical School, Boston, Massachusetts; 6 Caen University Hospital, University of Caen Normandy, Caen, France

## Abstract

This secondary analysis of a randomized clinical trial assesses patient-reported anxiety, depression, and quality of life among patients receiving mindfulness-based stress reduction vs escitalopram for the treatment of anxiety disorders.

## Introduction

Mindfulness-based interventions, such as mindfulness-based stress reduction (MBSR), have shown efficacy for anxiety disorders.^1^ We previously demonstrated that 8 weeks of MBSR was noninferior to escitalopram for treatment of anxiety disorders in a fully powered, multisite, noninferiority parallel-group randomized clinical trial with a predetermined margin.^2^ We present secondary outcomes of the trial, including patient-reported anxiety, depression, and quality of life (QOL).

## Methods

The trial included community-dwelling adults with a primary anxiety disorder (agoraphobia, panic, generalized anxiety, or social anxiety disorder) diagnosed by a clinician before 1:1 randomization.^2^ The MBSR intervention comprised weekly classes that included theory and practice of several forms of mindfulness meditation. Escitalopram recipients met with a prescriber at baseline and at weeks 1, 2, 4, 6, 8, and 12 and were flexibly dosed (10-20 mg/d). Local institutional review boards approved the trial protocol (Supplement 1), and patients provided informed consent. The CONSORT reporting guideline was followed.

Blinded evaluators assessed patient-reported (Beck Anxiety Inventory [BAI], PROMIS Anxiety Short Form, and Penn State Worry Questionnaire) and clinician-reported (Liebowitz Social Anxiety Scale, Panic Disorder Severity Scale, and Structured Interview Guide for the Hamilton Anxiety Scale) anxiety outcomes. Depression symptoms were assessed using the PROMIS Depression scale; QOL and role functioning were assessed with the PROMIS Satisfaction With Participation in Social Roles and the PROMIS Ability to Participate in Social Roles and Activities scales (eMethods in Supplement 2).^3^

Groups were compared using bivariate statistics and in multivariable regression analyses. Adjusted analyses used linear mixed models that included all participants at baseline regardless of missing data on other time points, according to intent-to-treat principle. Models were adjusted for sex, age, self-reported race and ethnicity (collected per National Institutes of Health requirements),^2^ baseline anxiety severity (low vs high), and site; included time and treatment indicators and their interactions; and were estimated with participant-level random effects. Between-group mean differences (MDs) were estimated for each time point. Analyses were conducted with Stata, version 15 (StataCorp LLC).

## Results

This analysis included 276 participants (207 women [75%] and 69 men [25%]; mean [SD] age, 33 [13] years) (eFigure in Supplement 2). Participants identified as Asian (51 [19%], Black (40 [15%]), Hispanic (25 [9%]), White (166 [60%]), or other race or ethnicity (19 [6%]).^2^ No significant between-group differences in demographic characteristics or baseline outcome measures were observed, except for BAI scores. Participants in both groups demonstrated reductions in anxiety symptoms (Table 1), with between-group differences corresponding to small effect sizes (Cohen *d* ≤ 0.20). Table 2 presents between-group estimated MDs at all time points; no significant differences were detected at week 8 (primary end point). At week 4 (midtreatment), significant differences in PROMIS Anxiety and PROMIS Depression scores emerged, with greater improvement with escitalopram, but were no longer significant at week 8. However, 110 escitalopram recipients (78.6%) had at least 1 study-related adverse event vs 21 MBSR recipients (15.4%) (Cohen *h* = 1.37 [95% CI, 0.54-0.72]; *P* < .001).

**Table 1.** Assessment of Anxiety Symptoms With MBSR and Escitalopram Treatment

Measure	MBSR (n = 136)		Escitalopram (n = 140)		Cohen *d* effect size^a^	*P* value^a^
	Mean score (SD)	No. of participants	Mean score (SD)	No. of participants		
**PROMIS Anxiety–T, 8-item version**						
Baseline	64.7 (5.8)	136	64.8 (5.7)	139	NA	.82
Primary end point: week 8	57.4 (7.9)	111	55.9 (7.7)	109	0.20	.15
Follow-up						
Week 12	56.1 (8.6)	104	55.5 (9.1)	99	0.07	.60
Week 24	56.3 (8.2)	99	55.2 (8.3)	101	0.13	.34
**BAI (range, 0-63)**						
Baseline	15.3 (8.9)	136	17.4 (9.1)	139	NA	.05^b^
Primary end point: week 8	8.5 (8.6)	111	7.8 (7.4)	109	0.09	.49
Follow-up						
Week 12	7.0 (7.4)	104	6.7 (6.4)	100	0.04	.78
Week 24	7.3 (6.3)	99	6.2 (5.6)	101	0.18	.22
**PROMIS Depression–T, version 8b**						
Baseline	58.3 (7.4)	136	57.6 (8.0)	139	NA	.46
Primary end point: week 8	52.7 (8.1)	111	51.4 (9.2)	109	0.15	.29
Follow-up						
Week 12	52.0 (9.2)	104	50.7 (9.4)	99	0.14	.31
Week 24	51.9 (8.7)	99	50.1 (9.2)	101	0.20	.16
**PSWQ (range, 16-80)**						
Baseline	64.1 (9.6)	130	65.3 (8.9)	134	NA	.31
Primary end point: week 8	53.9 (13.1)	111	51.7 (13.3)	109	0.17	.22
Follow-up						
Week 12	51.7 (13.6)	104	52.0 (14.4)	99	0.02	.90
Week 24	51.9 (12.7)	99	52.1 (13.3)	101	0.02	.92
**LSAS (range 0-144)**						
Baseline	60.0 (25.8)	135	63.6 (27.1)	140	NA	.26
Primary end point: week 8	39.9 (26.8)	112	40.9 (27.1)	111	0.04	.77
Follow-up						
Week 12	40.0 (28.1)	105	38.5 (29.0)	106	0.05	.70
Week 24	39.4 (28.4)	104	40.2 (26.8)	98	0.03	.84
**PDSS (range, 0-28)**						
Baseline	4.0 (4.8)	136	5.0 (5.7)	140	NA	.14
Primary end point: week 8	1.6 (3.0)	111	2.1 (3.5)	111	0.15	.26
Follow-up						
Week 12	1.5 (2.6)	105	2.1 (4.1)	106	0.17	.18
Week 24	1.6 (2.9)	104	1.9 (3.8)	98	0.09	.44
**SIGH-A**						
Baseline	19.2 (7.1)	136	20.5 (6.9)	140	NA	.13
Primary end point: week 8	11.6 (7.4)	111	11.6 (7.1)	109	0.01	.96
Follow-up						
Week 12	11.0 (7.3)	105	11.8 (7.0)	106	0.11	.42
Week 24	11.6 (7.5)	104	10.8 (6.8)	98	0.11	.39
**PROMIS-APSRA–T **						
Baseline	43.2 (4.9)	135	41.7 (5.1)	139	NA	.02^b^
Primary end point: week 8	46.5 (5.5)	110	46.2 (6.3)	107	0.04	.78
Follow-up						
Week 12	46.3 (5.7)	104	46.1 (6.6)	98	0.03	.85
Week 24	46.8 (6.0)	99	46.5 (5.8)	101	0.05	.75
**PROMIS-SPSR–T**						
Baseline	42.9 (6.3)	135	42.3 (6.0)	138	NA	.42
Primary end point: week 8	47.9 (6.8)	110	47.0 (8.7)	107	0.12	.39
Follow-up						
Week 12	48.6 (8.1)	104	48.1 (8.8)	98	0.06	.68
Week 24	48.0 (7.2)	99	46.3 (7.8)	101	0.23	.11

Abbreviations: APSRA, Ability to Participate in Social Roles and Activities; BAI, Beck Anxiety Inventory; LSAS, Liebowitz Social Anxiety; MBSR, mindfulness-based stress reduction; NA, not applicable; PDSS, Panic Disorder Severity Scale; PROMIS, Patient-Reported Outcomes Measurement Information System; PSWQ, Penn State Worry Questionnaire; SIGH-A, Structured Interview Guide for the Hamilton Anxiety Scale; SPSR, Satisfaction With Participation in Social Roles; T, *t* score.

^a^ Based on 2-tailed, 2-sample *t* tests between groups.

**Table 2.** Estimated Mean Differences Between Treatment Groups^a^

	Escitalopram vs MBSR, estimated mean difference (95% CI)								
	PROMIS Anxiety–T (n = 275)	BAI (n = 275)	PROMIS Depression–T (n = 275)	PSWQ (n = 273)	LSAS (n = 276)	PDSS (n = 276)	SIGH-A (n = 276)	PROMIS APSRA–T (n = 274)	PROMIS SPSR–T (n = 273)
Time point									
Baseline	−0.0 (−1.7 to 1.6)	2.0 (0.3 to 3.7)	−0.8 (−2.7 to 1.1)	0.8 (−1.9 to 3.6)	3.0 (−3.1 to 9.1)	0.9 (−0.0 to 1.9)	1.1 (−0.5 to 2.6)	−1.3 (−2.6 to −0.1)^b^	−0.4 (−2.1 to 1.2)
Week 4	−2.6 (−4.4 to −0.8)^c^	−1.4 (−3.3 to 0.5)	−3.8 (−5.9 to −1.8)^d^	−2.9 (−5.8 to 0.1)	−0.4 (−6.8 to 5.9)	0.1 (−0.9 to 1.1)	0.1 (−1.5 to 1.8)	−0.4 (−1.7 to 1.0)	0.1 (−1.7 to 1.9)
Week 8	−1.6 (−3.4 to 0.3)	−0.8 (−2.7 to 1.1)	−1.4 (−3.5 to 0.7)	−2.6 (−5.6 to 0.3)	1.3 (−5.1 to 7.6)	0.5 (−0.5 to 1.5)	−0.2 (−1.9 to 1.4)	−0.2 (−1.5 to 1.2)	−0.8 (−2.6 to 1.0)
Week 12	−1.1 (−3.0 to 0.8)	−0.7 (−2.6 to 1.2)	−1.8 (−3.9 to 0.3)	−0.4 (−3.4 to 2.7)	−0.7 (−7.1 to 5.7)	0.6 (−0.4 to 1.7)	0.4 (−1.3 to 2.1)	0.0 (−1.4 to 1.4)	−0.3 (−2.1 to 1.2)
Week 24	−1.3 (−3.2 to 0.6)	−1.2 (−3.1 to 1.2)	−2.1 (−4.3 to 0.0)^b^	−0.3 (−3.3 to 2.7)	2.6 (−3.8 to 9.1)	0.5 (−0.4 to 1.7)	−1.1 (−2.8 to 0.6)	−0.1 (−1.6 to 1.3)	−1.6 (−3.5 to 0.2)
No. of observations	1118	1120	1118	1108	1137	1137	1135	1113	1112
Wald χ^2^ (*df*)	520.9 (17)	627.8 (17)	297.9 (17)	512.4 (17)	625.9 (17)	204.0 (17)	683.8 (17)	242.3 (17)	247.2 (17)

Abbreviations: APSRA, Ability to Participate in Social Roles and Activities; BAI, Beck Anxiety Inventory; LSAS, Liebowitz Social Anxiety; MBSR, mindfulness-based stress reduction; PDSS, Panic Disorder Severity Scale; PROMIS, Patient-Reported Outcomes Measurement Information System; PSWQ, Penn State Worry Questionnaire; SIGH-A, Structured Interview Guide for the Hamilton Anxiety Scale; SPSR, Satisfaction With Participation in Social Roles; T, *t* score.

^a^ Based on linear mixed models of secondary outcome measures adjusted by age, sex, race and ethnicity, site, and severity with interactions between treatment group and time point indicators. A lower negative value demonstrates a lower total score in the escitalopram group.

^b^ *P* < .05.

^c^ *P* < .01.

^d^ *P* < .001.

## Discussion

In our previous trial, MBSR was noninferior to escitalopram based on a transdiagnostic clinician-rated primary outcome.^2^ To our knowledge, this secondary analysis is the first to describe patient-reported anxiety and depression outcomes and disorder-specific clinician-rated anxiety measures comparing mindfulness with pharmacotherapy. Overall, we were unable to detect significant between-group differences in outcomes, and the effect sizes were small (*d* = 0.01-0.20), suggesting a lack of clinically meaningful differences in effectiveness between treatments. At midtreatment but not study end, scores on a few measures indicated greater symptom reduction with escitalopram. Overall, our findings are consistent with previous work demonstrating the efficacy of mindfulness for panic and social anxiety disorders.^4^

This study has some limitations. Recipients of MBSR had more face-to-face time with their meditation instructor and prescriber. Additionally, noninferiority testing was not feasible with our sample size due to multiple secondary outcomes. Taken together, these findings support clinical application of MBSR to treat anxiety disorders, with outcomes similar to antidepressant pharmacotherapy but with potentially fewer side effects.

## Notes

Supplement 1.
Trial Protocol and Statistical Analysis Plan

Supplement 2.
eMethods
**eFigure.** CONSORT Flow Diagram

Supplement 3.
Data Sharing Statement
