# Supplementary material for: Mindfulness Meditation vs Escitalopram for Treatment of Anxiety Disorders: Secondary Analysis of a Randomized Clinical Trial
Source: JAMA Netw Open. 2024 Oct 9;7(10):e2438453. doi: 10.1001/jamanetworkopen.2024.38453 (PMC11581486; doi:10.1001/jamanetworkopen.2024.38453)
Supplement: Supplement 1. — Trial Protocol and Statistical Analysis Plan [file jamanetwopen-e2438453-s001.pdf]

Detailed Protocol
Version #26
April 20, 2022

Protocol: Comparative Effectiveness of Mindfulness-Based Stress Reduction and Pharmacotherapy for Anxiety

I. STUDY PERSONNEL

Principal Investigator: Elizabeth Hoge, MD
Department of Psychiatry, Research Division

Co-investigator: Mary Ann Dutton, PhD
Department of Psychiatry, Research Division

Co-investigator: Alfiee Breland-Noble, PhD
Department of Psychiatry, Research Division

Research Coordinator: Caroline Armstrong, BA

Biostatistician Mihriye Mete, PhD
Medstar Health Research Institute

II. FUNDING

This study is supported with a grant from Patient Centered Outcome Research Institute under the category of Assessment of Prevention Diagnosis and Treatment Options.

III. BACKGROUND AND SIGNIFICANCE

Anxiety disorders (generalized anxiety disorder, social anxiety disorder, panic disorder, and agoraphobia) are associated with significant distress and impairment in social and occupational functioning [1] and increased risk for suicide [2]. While pharmacotherapy and psychotherapy are first-line treatment strategies for anxiety disorders, many patients are reluctant to take psychiatric medication, and many prefer to avoid psychiatric care due to stigma or distrust of traditional medical care [3-6]. Pharmacotherapy approaches including selective serotonin reuptake inhibitors (SSRIs) have shown efficacy in treating anxiety disorders, however, side effects and stigma associated with psychotropic medications limit their adoption. Although Cognitive-Behavioral Therapy also has recognized efficacy, it is typically provided in a psychiatric clinic (which is associated with stigma) and is limited by availability of trained psychotherapists [7, 8]. Thus, it is increasingly important to compare the effectiveness of standard treatments to alternatives which are offered outside of a mental health context and may have better patient acceptability.

Mindfulness meditation training has seen an exponential growth in popularity in the general public, as illustrated by results from the NIH National Health Interview Survey finding that 18 million adults in the US have now tried meditation [9]. Patients with anxiety disorders are interested in mindfulness [8, 10]. However, although mindfulness meditation is commonly used to target anxiety [11], there is no information about how it compares to SSRI medication which is currently the most widely used treatment for anxiety disorders [12].

Individuals with anxiety disorders who are considering trying mindfulness do not currently have any evidence-based information to help them make decisions about how this new strategy compares to standard treatments. This proposal aims to fill that gap. Other stakeholders, such as those from healthcare systems and payers need this information to help guide decisions about integrating mindfulness interventions into existing treatment options. If mindfulness meditation was just as effective as SSRI medication, which is considered first-line treatment in the field, this would support insurance reimbursement and wider implementation of mindfulness meditation treatments across healthcare domains.

Mindfulness practice involves focusing on current thoughts, feelings, and physiological sensations, and thus, mindfulness practice trains a person to act consciously rather than react unconsciously to external or internal stimuli [13]. By continually practicing being "mindful," an individual learns to stay focused on present thoughts and emotions during stress, rather than getting distracted by anxiety-related sensations and worries, which can lead to better coping and more successful problem-solving behaviors [14]. Systematic mindfulness meditation training has been manualized in a group format called Mindfulness-Based Stress Reduction (MBSR). Several studies have already demonstrated positive findings for mindfulness meditation interventions in patients with anxiety [15-17]. In addition, our tightly-controlled, randomized trial of MBSR vs. an attention control in individuals with generalized anxiety disorder (35% with co-morbid anxiety disorders) yielded greater drops in several anxiety symptom scales assessed by blinded clinical raters (see *Preliminary Data* section below). Unfortunately, the critical lack of information about how mindfulness meditation training compares with existing treatments including SSRI medications, in terms of effectiveness and acceptability, prevents patients from being able to make informed decisions when choosing a treatment approach.

IV. SPECIFIC AIMS (Research Objectives)

We will assess the comparative effectiveness of Mindfulness-Based Stress Reduction (MBSR) compared to escitalopram (ESC) for patients with anxiety disorders (generalized anxiety disorder, panic disorder, agoraphobia, and social anxiety disorder), by randomizing patients to eight weeks of (1) MBSR or (2), flexible-dose escitalopram. This two-arm, three-site, randomized, controlled study will use a standard non-inferiority study design which incorporates previous data demonstrating that escitalopram is an effective treatment for anxiety and has been proven against pill placebo. Based on non-inferiority driven power calculations (see Analysis plan), we plan to randomize 368 patients to either MBSR or medication. We expect that 276 will complete the protocol after 25% dropout; however treatment satisfaction including dropout and intent-to-treatment outcomes will also be important assessments to guide conclusions about patient preferences and treatment responses. This non-inferiority randomized design with standardized blinded assessments provides the most rigorous comparison data regarding comparison of the novel treatment of MBSR to a gold standard treatment, escitalopram. This study will provide crucial information to patients about effective and acceptable treatment strategies for anxiety.

We will examine several secondary outcome measures and exploratory outcomes. Overall Anxiety Severity and Impairment Scale (OASIS) is the secondary measure of anxiety symptoms. To assess satisfaction/acceptability of treatment, we will use the Client Satisfaction Questionnaire (CSQ) and the Reactions to Treatment Questionnaire (RTQ). To assess sleep outcomes, we will use the Pittsburgh Sleep Quality Index (PSQI), The Beck Anxiety Inventory (BAI) is a 21-item self-report inventory designed to measure severity of anxiety symptoms in

psychiatric populations. The PROMIS - Emotional Distress scales (ED) will be used to assess emotional distress in the past 7 days, including depressive symptoms, anxiety symptoms, and anger. Other patient-reported measures include the Penn State Worry Questionnaire (PSWQ), and the Health Performance Questionnaire (HPQ). We will also use the Quick Inventory of Depressive Symptomatology, Self-Report, or QIDS-SR to measure depression severity including suicidality Other clinician-reported outcomes include the Liebowitz Social Anxiety Scale (LSAS), the Panic Disorder Severity Scale (PDSS), the Structured Interview Guide for the Hamilton Anxiety Scale (SIGH-A), the Clinician Global Impression-Severity and Improvement Scales (CGI-S and CGI-I), and the Frequency, Intensity, and Burden of Side Effects Ratings (FIBSER).

V. SUBJECT SELECTION

We aim to enroll 620 participants total across the three study sites (GUMC, NYU, and MGH Boston). With a projected dropout rate of 25%, we expect 465 completers. Subjects must be between 18-75 years of age, have an anxiety disorder, and agree to participate in this randomized controlled study in which they will be randomized to either an MBSR class or medication.

Inclusion Criteria:

1. Men and women between age 18 and 75 years old.
2. Have a primary anxiety disorder, including: social anxiety disorder (SAD), generalized anxiety disorder (GAD), panic disorder, or agoraphobia
3. Must understand study procedure and willing to participate in all testing visits, and treatment as assigned.
4. Participants must be able to give informed consent to the study procedures.

Exclusion Criteria:

1. Comorbid psychiatric disorder other than anxiety or depression, such as psychotic disorder, obsessive compulsive disorder, eating disorders (i.e., anorexia and bulimia), bipolar disorder; developmental or organic mental disorders; and current (past 6 months) substance use disorders and current post-traumatic stress disorder as assessed by clinician at screening visit
2. A serious medical condition that may result in surgery or hospitalization.
3. A history of head trauma causing prolonged loss of consciousness, or ongoing cognitive impairment
4. Inability to understand study procedures or informed consent process, or significant personality dysfunction likely to interfere with study participation (assessed during the clinical interview).
5. Subjects who will be non-compliant with the study procedures. This may include planned travel out of town.
6. Pregnancy as assessed by urine test at screen. Avoidance of pregnancy is also necessary for inclusion in this study.
7. Subjects taking barbiturates, SSRIs, anti-depressants, or antipsychotics. Sleep medications (other than anti-depressants) and benzodiazepines will be allowed, if has been taken at stable dose 4 weeks prior to baseline and the patient plans to continue at the same dose through the trial. Trazadone (for sleep) above 100mg will be disallowed.

8. Concurrent psychotherapy initiated within 1 month of screen interview, or ongoing psychotherapy of any duration directed specifically toward the treatment of anxiety (such as Cognitive Behavioral Therapy).
9. Individuals who have completed a course of MBSR or an equivalent meditation training in the last year, or have an ongoing daily meditation practice.
10. Individuals reporting significant active suicidal ideation or suicidal behaviors within the past year.
11. Individuals with a medical condition (i.e., epilepsy) that may be exacerbated by study treatment, as determined by a study physician or nurse practitioner based on history, physical, and/or labs.

VI. SUBJECT ENROLLMENT

All participants will be recruited from the general public to participate in a study investigating the effects of MBSR compared to escitalopram on anxiety disorders. Most participants are expected to be self-referred, treatment-seeking patients. In addition, patients will be recruited through: 1) word of mouth referrals, 2) referrals from area medical and mental health professionals at the three sites, 3) Patient partner-panel and IRB approved information about the study on our websites and through local approved postings, and 4) advertisements placed in local media. Potential participants who contact the research assistant will be screened for eligibility and interest in research participation. If interested and eligible for the study, they will meet with the study clinician who will obtain written informed consent after a thorough explanation of procedures including the treatment options along with a brief rationale for these approaches, and the potential risks involved in participation. The participant will be invited to ask questions which will be answered. Patients will be asked to sign the informed consent statement. The consent form will also state that participation is voluntary, that participants can refuse to answer any question, that they can withdraw from the study at any time, and that study participation in no way affects their care at GUMC, MGH, or NYU. The IRBs of GUMC, MGH, and NYU will have approved the protocol and consent forms prior to the initiation of the study. If the participant signs the consent form, they will be given a copy of the consent form regardless of whether they enroll in the study.

VII. STUDY PROCEDURES

Patients with anxiety disorders from nearby communities and in the surrounding metropolitan areas will be invited to participate through study recruitment materials. Participants may be engaged using IRB-approved print and radio advertisements and clinic referral at GUMC, NYU, and MGH. Participants may also be solicited through IRB-approved media, flyers, postings and other primary care and psychiatric clinic facilities and programs. Referrals may be made by medical and mental health practitioners in the area as well as self-referrals by affected individuals.

Individuals who contact the study coordinators at each site will be phone screened for general medical and diagnostic treatment eligibility and interest in research participation. If they are eligible and interested, they will then have a meeting with a study clinician to evaluate their psychiatric diagnosis and eligibility. This first visit (Screening Visit) includes a psychiatric (using the MINI interview) and medical assessment and brief physical exam, to determine eligibility. The study clinician will inquire whether the participant has a primary care clinician or psychiatrist who already did a medical work up to rule out medical causes of anxiety symptoms. If they have, staff will ask the participant to sign a release form in order to give permission to speak with the participant’s doctor or to provide the study team with a copy of past blood tests or ECG

or if neither a confirmation from records or the outside provider is not possible, study staff will perform an ECG and blood test.

For all participants, urine testing for pregnancy and drugs of abuse will be completed to help determine whether exclusion criteria are met (pregnancy, substance abuse) by the research coordinator. The Screening Visit will take about 3 hours. The study will be open to adults 18 – 75 years old who have an anxiety disorder; all races, and ethnicities and genders are invited. We will repeat the Screening Visit if extenuating circumstances lead to an abnormally long waiting time between the Screening Visit and Baseline Visit, in order to obtain the most accurate and up-to-date information.

The second study visit in the Baseline Assessment visit, which takes place within the 6-week period before the start of treatment. If unusual circumstances prevent the participant from beginning treatment within the 6-week window following the Baseline Assessment, the visit will be repeated to obtain the most accurate and up-to-date information. Patients will be assessed by the “Independent Evaluator” who will remain blind to treatment allocation, and who will complete the clinician-rated instruments (please see Table of Assessments). Assessments will be made by trained, independent evaluators (IE's) either in person, by videoconferencing, or by phone. If assessments are competed by videoconferencing or phone due to concerns of coronavirus, we will omit collecting vital signs and weight at that visit. All IEs for this multicenter trial will be undergoing shared training and supervision across sites. If necessary, IE's can assess patients at any of the study sites. Patients will be asked to complete a series of patient-reported questionnaires (please see table that consist of questions about the participants’ thoughts, feelings, anxiety symptoms, depression symptoms, adverse and traumatic experiences in the past, sleep, and other topics; these will be repeated in subsequent study visits (see Table of Assessments).

<u>Table of Assessments</u>

Study Week	Screen	Baseline Assessment visit	Week 1	Week 2	Week 4	Week 6	Primary Endpoint visit	Follow-up: Week 12	Follow-Up: Week 24
Patient-reported measures of outcome									
Demographics	✖								
Anxiety (OASIS anxiety scale)		✖	✖	✖	✖	✖	✖	✖	✖
Anxiety (BAI)		✖			✖		✖	✖	✖
Worry (PSWQ)		✖			✖		✖	✖	✖
Emotion Regulation (DERS-SF)		✖			✖		✖		
Self-Compassion Scale (SCS)		✖			✖		✖		
Emotional distress (PROMIS-Depression SF and PROMIS-Anxiety SF)		✖			✖		✖	✖	✖
Treatment Satisfaction (CSQ, RTQ)			✖		✖		✖	✖	
Sleep (PSQI, PROMIS-sleep anxiety measure)		✖			✖		✖		✖
Quality of Life/Functioning		✖			✖		✖	✖	✖

(PROMIS- SPSR and PROMIS- APSRA-8)									
Adverse Experiences (CTQ and LEC)	✖								
Work performance (HPQ)		✖							✖
Clinician-rated assessments of outcome									
Psychiatric Diagnoses (MINI)	✖								
Anxiety symptom severity (CGI-S /CGI-I)		✖			✖		✖	✖	✖
Disorder-specific scales (PDSS, SIGH-A, LSAS)		✖			✖		✖	✖	✖
Measures of Safety and Study Integrity									
Side Effects (FIBSER & Adverse Events)			✖	✖	✖	✖	✖	✖	✖
Adherence/ Homework			✖	✖	✖	✖	✖	✖	✖
Depressive symptoms (QIDS-SR including suicidality item)		✖			✖		✖	✖	
Vital Signs	✖				✖		✖		
Clinician Suicide Assessment	✖	✖*	✖	✖	✖	✖	✖	✖	✖
Concomitant Treatment	✖	✖*	✖	✖	✖	✖	✖	✖	✖
Weight	✖				✖		✖		
Blood draw, ECG, Urine pregnancy/drug test**	✖								

*these measures will be repeated with MD/NP during first week of treatment (referred to as "week 0") <u>unless</u> the participant has been randomized to MBSR *and* less than two weeks elapse between that participant's baseline visit and the start of their treatment)
**these procedures will only be completed if pre-existing records are not obtainable

Participants will be paid a total of $160 participation in the study. If a patient misses a treatment visit, the provider (MBSR instructor or Medical Rater) will reach out to them and work with the patient to minimize any barriers. For all assessment visits, including the follow-up visits at 12 and 24 weeks, we will offer flexible visits which can be done over the phone, videoconferencing, and/or electronically, if needed. In order to reduce missing data, as well as reducing the likelihood that treatment related dropout biases the available data for this effectiveness study in which treatment satisfaction, tolerability, adherence and response are all clinically relevant, to the extent feasible, we will follow subjects who miss treatment visits or drop out of the assigned protocol (either voluntarily, or removed by the study due to adverse effects) and continue to collect their assessment data and include them in the analysis according to their original assignment.

<u>Follow-Up at 24 Weeks</u>

The final study visit will occur at 24 weeks, which will include clinician-rated and patient-rated study measures. After the last study visit at 24 weeks, participants will be offered the opportunity to try the treatment in the opposite study arm (the one they were not randomized to), or, if

initially randomized to escitalopram, to continue on the medication, if preferred. We believe that these extra, post-study treatment offerings will make participants more likely to enroll in the trial, since they will know that they will have the chance to try the treatment to which they were not randomized. Participants in the escitalopram arm who wish to try MBSR will be offered a spot in a class at no cost. Participants in the MBSR arm who wish to try escitalopram will be seen by a study MD/NP who will monitor medication effects for 2 months, after which time the patient will be referred back to his/her primary care provider or other clinician for continuation of care, if the patient desires to stay on the medication.

Treatments

Mindfulness-Based Stress Reduction (MBSR)

MBSR is an 8-week group-based course developed by Jon Kabat-Zinn (1990) and colleagues at the University of Massachusetts' Center for Mindfulness. Weekly 2.5-hour long classes are given once a week, as well as one day-long weekend class during the 6th week. The classes instruct participants in the theory and practice of several forms of mindfulness meditation: a body scan (bringing awareness through the body systematically); breathing awareness (attention focused on the breath and other physical sensations); and mindfulness stretching exercises designed to bring awareness of the body and current experience of movement. Didactic teaching of the theory of mindfulness and experiential practice are both utilized during weekly classes and at-home CD-guided practice sessions.

If the MBSR course is canceled mid-way through, or is converted to an online platform for any reason (e.g., coronavirus), participants will be permitted to enroll in the online version of MBSR, or will be enrolled in a later course to finish their treatment.

Escitalopram (ESC)

During the 8 weeks of randomized treatment with ESC, subjects will be seen at baseline, post-baseline medication pick-up visit, weeks 1, 2, 4, 6, and 8 week endpoint, and at 12 week follow-up, (9 visits total) by a study physician/nurse practitioner. Georgetown University Medical Center's research pharmacy will be responsible for preparing the medication, which will be given to the participants by a study M.D. from baseline to week 12. Patients will take the pills each day at home. ESC will be initiated at 10 mg/day; which will be continued to week 2, when it will be increased to 20mg/day if well tolerated (or delayed if not). Medication adherence will be assessed by pill count and a standardized adherence measurement [68]. Side effects will be assessed at each visit and recorded. Participants will also be instructed to contact study staff immediately if any significant side effects, symptomatic worsening, or suicidal ideation develops in between visits. After week 12, if the patient desires to stay on the medication, s/he will be given a prescription by the study MD/NP., who will also start the process of transition to outpatient care so that the patient can continue treatment after the end of the study, if desired.

If in-person visits are not possible for any reason (e.g., coronavirus), the study medication will be shipped directly to the patient from the research pharmacy following the pharmacy protocols and the study visits will conducted using telehealth videoconferencing or telephone contact.

Adherence Documentation

Subjects’ attendance in MBSR classes will be recorded and subjects will record the number of minutes spent practicing with the free Google StopWatch application, or another time log application, or on paper, and will report the number of minutes to the research coordinator. Medication adherence will be assessed by pill count and a standardized adherence measurement [64].

Instruments and Scales

Patient-Reported Outcomes

Overall Anxiety Severity and Impairment Scale (OASIS) is the primary patient-reported measure of anxiety symptoms. The OASIS is a 5-item, self-report measure used to assess overall anxiety levels across diagnostic groups. Items are rated on a likert scale from 0-4, with higher values indicating greater clinical anxiety symptom severity. The OASIS has demonstrated good validity and reliability transdiagnostically in psychiatric samples of individuals with anxiety disorders, including patients with primary GAD, SAD, or PD [30-32]. This measure has been utilized as the primary outcome measure for various randomized control trials investigating treatment effectiveness across GAD, SAD, and PD patient populations [33, 34].

To assess satisfaction/acceptability of treatment, we will use the **Client Satisfaction Questionnaire** (CSQ) [35] and the **Reactions to Treatment Questionnaire** (RTQ) [36]. To assess sleep outcomes, we will use the **Pittsburgh Sleep Quality Index (PSQI),** a 24-item patient administered scale with demonstrated validity and reliability assessing subjective sleep quality [37], as well as the **PROMIS Sleep-Anxiety measure**, which consists of 13 items from the PROMIS Sleep Disturbance battery, that relate specifically to sleep disturbance resulting from worry, anxiety, or stress .

The Beck Anxiety Inventory (BAI) is a 21-item self-report inventory designed to measure severity of anxiety symptoms in psychiatric populations has high internal consistency, established validity, and test-retest reliability [38]. The BAI has both a cognitive and somatic subscale and has been used for over 20 years.

The **Difficulties in Emotion Regulation Scale**- Short Form (Kaufman et al. 2015) is an 18-item self-report measure that has established validity and is widely used for assessing emotion regulation style in adults.

The **Self-Compassion Scale (SCS)** [64] is a 26-item self-report instrument that uses a 5-point Likert scale for each item to assess compassion and kindness towards oneself, partially through seeing one’s experiences as not unique and specific to the individual, but part of a larger human experience. The SCS has six subscales: Self-Kindness, Common Humanity, Mindfulness, Self-Judgment, Isolation, and Over-Identification. The SCS has demonstrated good reliability and validity in English and several other languages.

The PROMIS - Emotional Distress scales (ED) [39] will be used to assess emotional distress in the past 7 days, including depressive symptoms, anxiety symptoms, and anger. Another patient-reported outcome of anxiety, and worry in particularly, is the Penn State Worry

Questionnaire (PSWQ) [40]. We will also use the Quick Inventory of Depressive Symptomatology, Self-Report, or QIDS-SR [41] to measure depression severity including suicidality.

To assess quality of life, we will use the **PROMIS - Satisfaction with Participation in Social Roles** - Short Form (SPSR) [42] that assess contentment with social roles, such as work and family responsibilities, within the past 7 days, and **the PROMIS - Ability to Participate in Social Roles and Activities - Short Form 4A** (APSRA) [42] that assess participants' perceived ability to perform their usual social roles and activities.

Adherence for the MBSR arm will be measured by class participation and a self-report Treatment Tracking log in REDCap, where participants will report the number of minutes that they spent meditating each day over the past week. Participants will be asked to track their meditation minutes using the free Google StopWatch application, or another time log application, or on paper.

The Childhood Trauma Questionnaire (**CTQ-28**) is a 28 item self-report questionnaire that assesses patients' history of childhood abuse and neglect and specifically focuses on emotional abuse, physical and emotional neglect, and sexual abuse. [61]

The **Life Events Checklist** (LEC) consists of a list of 17 items describing traumatic experiences (fire, exposure to toxic substances, etc), each which is checked off as "Happened to me", "Witnessed it", "Learned about it," "Part of my job," "Not sure," or "Doesn't apply." [62]

The **Health Performance Questionnaire (HPQ)** is a self-report survey measure of job performance [50]. The HPQ quantifies measures such as sickness absence and work performance. The HPQ is a valid and reliable tool for estimating the indirect workplace costs of illness [51]. It is also sensitive to changes [51] and when used longitudinally, the HPQ can reveal the cost-effectiveness of treatment.

The **Quick Inventory of Depressive Symptomatology, Self-Report (QIDS-SR)** is a 16-item self-report questionnaire that assesses depressive symptoms, including suicidality [41]. This tool is widely used and has established validity.

The **Adherence Questionnaire (AQ)** is a 2-item questionnaire that is used to determine what proportion of the time between visits the participant took their study medication as recommended, and to establish the reason(s) for deviating from the recommended dose (e.g., forgot, side effects, thought not needed, etc.).

<u>Clinician-Rated Outcomes</u>

The primary measure of anxiety symptoms is the **Clinician Global Impression-Severity Scale (CGI-S).** Additionally, we will utilize the **Clinical Global Impression- Improvement (CGI-I)** Scale [45-48]. The CGI has excellent psychometric properties, is treatment sensitive, and highly correlated with anxiety and depression scales across disorders [49].

The **MINI International Neuropsychiatric Interview MINI** will be administered by a trained interviewer, and will take approximately 25 minutes. This short, diagnostic structured interview

has proven to provide reliable diagnoses (Lecrubier et al., 1997) and is considerably shorter than other structured diagnostic interview tools.

The **Structured Interview Guide for the Hamilton** Anxiety Scale (**SIGH-A**) is designed to assist clinicians in evaluating anxiety symptoms through the HAM-A. The 14-item HAM-A (Hamilton 1959) was developed to assess general anxiety symptoms in a clinical population and has proven sensitive to change with treatment. The SIGH-A provides specific and detailed rating guidelines for the purposes of administering and scoring the HAM-A, and has demonstrated high interrater and test-retest reliability [59].

The Panic Disorder Severity Scale (PDSS) is a semi-structured interview of panic symptom severity. It contains 7 items assessing multiple dimensions of PD severity, including a) frequency of panic attacks, b) distress during panic attacks, c) anticipatory anxiety, d) agoraphobic fear and avoidance, e) interoceptive fear and avoidance, f) impairment of work and social functioning. The PDSS is a widely applied measure of the severity of panic disorder symptoms [58].

The **Liebowitz Social Anxiety Scale** (**LSAS**) is a reliable and validated measure of social anxiety, that assesses both fear and anxiety about common social situations, as well as avoidance components of social anxiety [60].

The **Frequency, Intensity, and Burden of Side Effects Ratings (FIBSER)** is a 3-item, validated measure to assess the frequency, intensity, and interference of side effects due to study treatment. The **Adverse Events Form** is a form for the study clinician to fill out while interviewing patient which lists all adverse events and side effects due to study treatment.

The **Concomitant Treatment Form** is a form for the study clinician to fill out while interviewing patient which indicates forms of treatment that the patient may have started or stopped.

The **Clinician Suicide Assessment Form** is a form for the study clinician to fill out to assess the severity of a patient's suicidal ideation.

VIII. BIOSTATISTICAL ANALYSIS

Basic Statistical Analyses:
Initial statistical analysis will provide descriptive statistics on the demographic and clinical characteristics of the study participants using means, standard deviations, median and range for continuous variables and frequencies and percentages for categorical variables. To examine potential differences between the groups at baseline, descriptive statistics will be obtained for each group and compared statistically using two-sample t-tests, nonparametric rank tests, chi-square and Fisher's exact test as appropriate. All outcome variables will be summarized using similar methods for pre and post-treatment and 12 week follow-up.

Outcome Assessment:
The treatment response (CGI-I) will be compared by groups and tested via confidence intervals to examine the precision of non-inferiority. A review of methods for non-inferiority studies on behavioral treatments (Steinert et al 2017) found that of the eight published studies reviewed, the effect size used in the non-inferiority margin calculation ranged between d=0.26 and d=0.6. One of the examples in this review was a study of CBT that included all anxiety disorders,

similar to the sample in our current proposal (Norton and Barrera 2012). For this study, the researchers also surveyed the literature to determine the best way to calculate a non-inferiority margin with a transdiagnostic sample. They proposed that the non-inferiority margin be computed by multiplying the standard deviation of the particular outcome measure by the meta-analytically derived effect size, deriving their approach from a standard methodology recommended for rigorous equivalence designs (e.g. see Jones et al 1996). Using this calculation method, and using the SD of 1.1 points for CGI change scores from other escitalopram trials across anxiety disorders (see summary in Bandelow et al 2006), would have yielded a non-inferiority margin of 0.66 unit on the CGI-S (by multiplying 1.1 by 0.6). However, to be more conservative, a non-inferiority margin of 0.33 (0.3*1.1) was used in our sample size calculations. This margin represents a difference of 1/3 of a point on the CGI-S score. We also defined a largest-clinically-acceptable non-inferiority margin following the literature by determining the halfway point between these two numbers (0.66 and 0.33) to get 0.495, (0.45*1.1) such that a difference of 0.495 or smaller will still allow us to declare MBSR to be noninferior to escitalopram treatment. Further support for the appropriateness of this margin comes from the literature where a change of 1.0 unit, substantially higher than a margin of 0.495, is considered the Minimal Clinically Important Change (MCID) score for the CGI-S and has been used to anchor definitions of clinically significant change across other measures.

Therefore, the null hypothesis for the non-inferiority test will be that the drug group's mean CGI-S score reduction at endpoint is greater than that of the MBSR group by at least 0.495 points (for this scale, higher scores indicate higher severity and greater reduction indicated greater improvement). In other words, if the MBSR group's post-intervention mean CGI-S score change is less than that of the drug group by not more than 0.495 points we will consider MBSR to be non-inferior to the standard pharmacotherapy escitalopram. Based on these parameters, the required analysis sample size with equal allocation to achieve an 80% power at significance level of 0.05 will thus be N=276 study completers.

To conservatively address attrition, we estimate our projected dropout rate to be 25% and therefore plan to randomize 368 participants with a 1:1 ratio to each group, yielding 276 completers after drop-out. Of note, many published non-inferiority trials have relied on intent-to-treat analyses that are more liberal than our proposed completer analysis. We also propose to conduct sensitivity analyses with the intent-to-treat sample as a follow-up to our primary completer analysis.

Multivariate Analyses:
We will conduct secondary/advanced analyses by specifying multivariate and longitudinal regression models of the primary and secondary outcome measures, in order to estimate treatment differences adjusted by potential confounders, and to explore individual and group trajectories using available repeated measures data. Final analyses will include multi-level linear mixed-effect models and generalized linear and latent mixed models (GLLAMM) of the primary and secondary outcome measures by combining the data across all time points depending on the measurement scale of the dependent variables. We will include variables such as age, gender, race/ethnicity, education, income, diagnosis and severity in these models as confounders or moderators or control variables. The statistical evaluation of the coefficient estimate for the MBSR intervention indicator (average difference between the groups across all time points) will be based on both the confidence interval and p-value approaches described earlier (see Outcome Assessment) to assess the non-inferiority hypothesis in the context of generalized linear mixed effect models.

For secondary outcomes, data will be summarized using descriptive statistics such as means and standard deviations and frequencies and percentages for the overall sample and by treatment groups. Group differences will be first tested using bivariate statistics like t-tests and chi-square tests as appropriate. Secondary/advanced analyses will be conducted by specifying multivariable longitudinal regression models of the secondary outcome measures in order to estimate treatment differences at each time point while adjusting by potential confounders, and to explore individual and group trajectories using available repeated measures data. Linear mixed models will be adjusted for sex, age, race, anxiety severity at baseline (low vs high) and site, including time and treatment indicators and their interactions and estimated with random effects at participant level.

IX. RISKS AND DISCOMFORTS

Potential risks are: (i) loss of confidentiality, (ii) potential feelings of distress while completing assessments, (iii) potential feelings of distress while participating in MBSR, (iv) audio recording,(v) side effects of the escitalopram, and (vi) discomfort from the blood collection for screening labs.

(i) Loss of confidentiality: A potential risk is the loss of confidentiality. In order to protect confidentiality, data will be identified only by subject codes. The identity of patients will not be revealed in the presentation or publication of any results from the project. This study will be conducted in compliance with HIPAA and IRB guidelines on privacy and confidentiality in order to strictly respect and safeguard participants' confidentiality. All identifying data will be stored in locked or password protected files only accessible by study staff. Our research centers are very experienced in protecting patient confidentiality and we believe the risk of loss of confidentiality to be very small.

(ii) Assessments: One risk is the evocation of uncomfortable levels of stress, anxiety or other emotions during some assessments or questions. Participants will be informed about these risks and told that they may withdraw from the study at any time and may refuse to complete any questionnaire they find too uncomfortable. Study clinicians will also be available to speak with any patients who report distress during completion of any of the assessments.

(iii) MBSR: Some participants may find MBSR treatment sessions stressful and feel anxiety.. In addition, subjects may experience some interference with daily activities due to scheduling of treatment and assessment sessions. There is a risk of inactive or ineffective treatment. Participants will be informed about these risks and told that they may withdraw from the study at any time.

(iv) Audio Recordings: Some participants may also feel uncomfortable about having the MBSR group classes or study clinician sessions audiotaped and reviewed by others (necessary for treatment adherence checks and integrity of study procedures). The purpose of the taping will be explained, confidentiality will be maintained, and informed consent for taping will be obtained.

(v) Escitalopram:

Escitalopram is a widely available, safe and well-tolerated SSRI antidepressant that is FDA-approved for the treatment of Generalized Anxiety Disorder and commonly used for other anxiety disorders. The risks and discomforts associated with escitalopram are as follows: dry mouth, nausea, diarrhea, sweating, drowsiness, trouble sleeping, problems with sex, allergic

reaction (itching or hives, swelling in face or hands, swelling or tingling in the mouth or throat, tightness in chest, trouble breathing), confusion, bleeding, hepatic impairment, or weakness.

The most commonly observed adverse reactions in escitalopram oxalate patients (incidence of approximately 5% or greater and approximately twice the incidence in placebo patients) were nausea, ejaculation disorder (primarily ejaculatory delay), insomnia, fatigue, decreased libido, and anorgasmia.

A rare but serious adverse event Serotonin Syndrome, a potentially life-threatening syndrome that has been reported with SNRIs and SSRIs, including escitalopram oxalate, alone but particularly with concomitant use of other serotonergic drugs (including triptans, tricyclic antidepressants, fentanyl, lithium, tramadol, tryptophan, buspirone, amphetamines, and St. John's Wort) and with drugs that impair metabolism of serotonin (in particular, MAOIs, both those intended to treat psychiatric disorders and also others, such as linezolid and intravenous methylene blue). Serotonin syndrome symptoms may include mental status changes (e.g., agitation, hallucinations, delirium, and coma), autonomic instability (e.g., tachycardia, labile blood pressure, dizziness, diaphoresis, flushing, hyperthermia), neuromuscular symptoms (e.g., tremor, rigidity, myoclonus, hyperreflexia, incoordination) seizures, and/or gastrointestinal symptoms (e.g., nausea, vomiting, diarrhea). Patients will be informed about these symptoms and instructed to seek immediate medical attention if they occur.

In addition, there is a risk of increased suicidality with initiation of SSRI's in people with depression age 24 and under: subjects in this protocol will receive close monitoring for the development of suicidality (see Procedures to Minimize Risk in section 9.3). Escitalopram may also cause unexpected side effects. Adverse events (side effects) will be assessed by the study clinician. Participants will be instructed to contact the study physician/nurse practitioner should any adverse events or concerning side effects occur; if appropriate, the study doctor will refer him/her to treatment. At present there is insufficient information regarding the safety of escitalopram during pregnancy or breastfeeding. The procedures used in this study may be unsafe for an unborn baby. If you, as a subject of study, are a woman of child bearing potential, you must agree to avoid pregnancy during your participation in this study.
For more information about risks, contact the principal investigator at 202-687-0635.

If you do become pregnant during the study, you should immediately notify Dr. Hoge at 202-687-0635. In addition, if you are already pregnant, you cannot participate in this study
There are risks to suddenly stopping escitalopram including flu-like symptoms, headaches, and parathesias. These can be uncomfortable but are not dangerous.

(vi) Blood draw (for screening labs):

When blood is drawn, patients may feel some pain and have some bruising and/or bleeding at the needle site. Occasionally, a person feels faint when blood is drawn. Rarely, an infection, which can be treated, may develop at the injection site.

X. POTENTIAL BENFITS

Treatment may help to reduce physical or mental stress and decrease anxiety symptoms. Participants will receive $160 for their participation in each study visit. Other general benefits include yielding generalizable knowledge about the physiological effects of MBSR.

XI. REFERENCES

1. Ormel, J., et al., *Common mental disorders and disability across cultures. Results from the WHO Collaborative Study on Psychological Problems in General Health Care.* JAMA, 1994. **272**(22): p. 1741-8.
2. Nepon, J., et al., *The relationship between anxiety disorders and suicide attempts: findings from the National Epidemiologic Survey on Alcohol and Related Conditions.* Depress Anxiety, 2010. **27**(9): p. 791-8.
3. Givens, J.L., et al., *Older patients' aversion to antidepressants. A qualitative study.* J Gen Intern Med, 2006. **21**(2): p. 146-51.
4. Mitchell, A.J. and T. Selmes, *Why don't patients take their medicine? Reasons and solutions in psychiatry.* Advances in Psychiatric Treatment, 2007. **13**(5): p. 336-346.
5. Priest, R.G., et al., *Lay people's attitudes to treatment of depression: results of opinion poll for Defeat Depression Campaign just before its launch.* BMJ, 1996. **313**(7061): p. 858-9.
6. various. *Social Anxiety Support*. 2017 May 10, 2017]; Available from: http://www.socialanxietysupport.com/.
7. Shafran, R., et al., *Mind the gap: Improving the dissemination of CBT.* Behav Res Ther, 2009. **47**(11): p. 902-9.
8. AnxietyForum. *PLEASE cognitive behavioural therapy CURE OR NOT??* 2011 April 10, 2017]; Available from: http://anxietyforum.net/forum/showthread.php?8478-PLEASE-cognitive-behavioural-therapy-CURE-OR-NOT&highlight=anxiety+cognitive.
9. Clarke, P., C. MacLeod, and N. Shirazee, *Prepared for the worst: readiness to acquire threat bias and susceptibility to elevate trait anxiety.* Emotion, 2008. **8**(1): p. 47.
10. NoMorePanic. *MINDFULNESS discussion FORUM.* 2007 April 10, 2017]; Available from: http://www.nomorepanic.co.uk/showthread.php?t=20864.
11. Goyal, M., et al., *Meditation programs for psychological stress and well-being: a systematic review and meta-analysis.* JAMA internal medicine, 2014. **174**(3): p. 357-368.
12. Tyrer, P. and D. Baldwin, *Generalised anxiety disorder.* Lancet, 2006. **368**(9553): p. 2156-66.
13. Kang, Y., J. Gruber, and J.R. Gray, *Mindfulness and De-Automatization.* Emotion Review, 2013. **0**(0): p. 1-10.
14. Kabat-Zinn, J., *Full Catastrophe Living: Using the Wisdom of Your Body and Mind to Face Stress, Pain, and Illness*. 1990: Bantam Dell.
15. Vollestad, J., B. Sivertsen, and G.H. Nielsen, *Mindfulness-based stress reduction for patients with anxiety disorders: evaluation in a randomized controlled trial.* Behav Res Ther, 2011. **49**(4): p. 281-8.
16. Kim, Y.W., et al., *Effectiveness of mindfulness-based cognitive therapy as an adjuvant to pharmacotherapy in patients with panic disorder or generalized anxiety disorder.* Depression and anxiety, 2009. **26**(7): p. 601-606.
17. Evans, S., et al., *Mindfulness-based cognitive therapy for generalized anxiety disorder.* J Anxiety Disord, 2008. **22**(4): p. 716-21.
18. Ospina, M.B., et al., *Meditation practices for health: state of the research.* Evid Rep Technol Assess (Full Rep), 2007. **155**(155): p. 1-263.
19. Sox, H.C. and S. Greenfield, *Comparative effectiveness research: a report from the Institute of Medicine.* Annals of Internal Medicine, 2009. **151**(3): p. 203-205.
20. Purohit, M.P., et al., *Neuropsychiatric symptoms and the use of complementary and alternative medicine.* PM&R, 2013. **5**(1): p. 24-31.

21. Kessler, R.C., et al., *The use of complementary and alternative therapies to treat anxiety and depression in the United States.* American Journal of Psychiatry, 2001. **158**(2): p. 289-294.
22. YahooAnswers. *Search: "Alternative Treatments for Anxiety"*. 2017 2/10/17]; Available from: https://answers.search.yahoo.com/search?fr=uh3_answers_vert_gs&type=2button&p=alternative%20treatments%20to%20anxiety.
23. Various. *Psychology and Mental Health Forum*. 2017 2/10/17]; Available from: http://www.psychforums.com/.
24. Hoge, E.A., et al., *Randomized controlled trial of mindfulness meditation for generalized anxiety disorder: effects on anxiety and stress reactivity.* The Journal of clinical psychiatry, 2013. **74**(8): p. 786.
25. Krisanaprakornkit, T., et al., *Meditation therapy for anxiety disorders.* Cochrane Database Syst Rev, 2006(1): p. CD004998.
26. Hofmann, S.G., et al., *The effect of mindfulness-based therapy on anxiety and depression: A meta-analytic review.* J Consult Clin Psychol, 2010. **78**(2): p. 169-83.
27. Carlson, L.E., et al., *Mindfulness-based stress reduction in relation to quality of life, mood, symptoms of stress, and immune parameters in breast and prostate cancer outpatients.* Psychosom Med, 2003. **65**(4): p. 571-81.
28. Segal, Z.V., et al., *Antidepressant monotherapy vs sequential pharmacotherapy and mindfulness-based cognitive therapy, or placebo, for relapse prophylaxis in recurrent depression.* Arch Gen Psychiatry, 2010. **67**(12): p. 1256-64.
29. First, M., et al., *Structured Clinical Interview for DSM-5 Disorders–Research Version (SCID-5-RV).* Arlington: American Psychiatric Assocation, 2014.
30. Bragdon, L.B., et al., *Psychometric properties of the Overall Anxiety Severity and Impairment Scale (OASIS) among psychiatric outpatients.* Journal of affective disorders, 2016. **201**: p. 112-115.
31. Moore, S.A., et al., *Psychometric evaluation of the Overall Anxiety Severity And Impairment Scale (OASIS) in individuals seeking outpatient specialty treatment for anxiety-related disorders.* Journal of affective disorders, 2015. **175**: p. 463-470.
32. Norman, S.B., et al., *Development and validation of an overall anxiety severity and impairment scale (OASIS).* Depression and anxiety, 2006. **23**(4): p. 245-249.
33. Muntingh, A., et al., *Effectiveness of collaborative stepped care for anxiety disorders in primary care: a pragmatic cluster randomised controlled trial.* Psychotherapy and psychosomatics, 2014. **83**(1): p. 37-44.
34. Roy-Byrne, P., et al., *Delivery of evidence-based treatment for multiple anxiety disorders in primary care: a randomized controlled trial.* Jama, 2010. **303**(19): p. 1921-1928.
35. Attkisson, C.C. and R. Zwick, *The client satisfaction questionnaire. Psychometric properties and correlations with service utilization and psychotherapy outcome.* Eval Program Plann, 1982. **5**(3): p. 233-7.
36. Holt, C. and R. Heimberg, *The Reaction to Treatment Questionnaire: Measuring treatment credibility and outcome expectancies.* The Behavior Therapist, 1990. **13**(213-214): p. 222.
37. Buysse, D.J., et al., *The Pittsburgh Sleep Quality Index: a new instrument for psychiatric practice and research.* Psychiatry Res, 1989. **28**(2): p. 193-213.
38. Beck, A.T., et al., *An inventory for measuring clinical anxiety: psychometric properties.* J Consult Clin Psychol, 1988. **56**(6): p. 893-7.
39. Pilkonis, P.A., et al., *Item banks for measuring emotional distress from the Patient-Reported Outcomes Measurement Information System (PROMIS®): depression, anxiety, and anger.* Assessment, 2011. **18**(3): p. 263-283.

40. Meyer, T.J., et al., *Development and validation of the Penn State Worry Questionnaire.* Behav Res Ther, 1990. **28**(6): p. 487-95.
41. Rush, A.J., et al., *The 16-Item Quick Inventory of Depressive Symptomatology (QIDS), clinician rating (QIDS-C), and self-report (QIDS-SR): a psychometric evaluation in patients with chronic major depression.* Biological psychiatry, 2003. **54**(5): p. 573-583.
42. Hahn, E.A., et al., *Measuring social health in the patient-reported outcomes measurement information system (PROMIS): item bank development and testing.* Quality of Life Research, 2010. **19**(7): p. 1035-1044.
43. Leung, A.W. and R.G. Heimberg, *Homework compliance, perceptions of control, and outcome of cognitive-behavioral treatment of social phobia.* Behaviour Research and Therapy, 1996. **34**(5): p. 423-432.
44. Primakoff, L., N. Epstein, and L. Covi, *Homework compliance: An uncontrolled variable in cognitive therapy outcome research.* Behavior Therapy, 1986. **17**(4): p. 433-446.
45. Gross, P.K., R. Nourse, and T.E. Wasser, *Ramelteon for insomnia symptoms in a community sample of adults with generalized anxiety disorder: an open label study.* J Clin Sleep Med, 2009. **5**(1): p. 28-33.
46. Guy, W., *ECDEU assessment manual for psychopharmacology* 1976, US Department of Health, Education and Welfare Washington, DC. p. 217-222.
47. Varia, I. and F. Rauscher, *Treatment of generalized anxiety disorder with citalopram.* Int Clin Psychopharmacol, 2002. **17**(3): p. 103-7.
48. Worthington III, J.J., et al., *Aripiprazole as an augmentor of selective serotonin reuptake inhibitors in depression and anxiety disorder patients.* International clinical psychopharmacology, 2005. **20**(1): p. 9-11.
49. Zaider, T.I., et al., *Evaluation of the clinical global impression scale among individuals with social anxiety disorder.* Psychological medicine, 2003. **33**(04): p. 611-622.
50. Kessler, R.C., et al., *The world health organization health and work performance questionnaire (HPQ).* Journal of Occupational and Environmental Medicine, 2003. **45**(2): p. 156-174.
51. Kessler, R.C., et al., *Using the World Health Organization Health and Work Performance Questionnaire (HPQ) to evaluate the indirect workplace costs of illness.* Journal of Occupational and Environmental Medicine, 2004. **46**(6): p. S23-S37.
52. Hoge, E.A., et al., *Effects of mindfulness meditation on occupational functioning and health care utilization in individuals with anxiety.* J Psychosom Res, 2017. **95**: p. 7-11.
53. Harris, P.A., et al., *Research electronic data capture (REDCap)--a metadata-driven methodology and workflow process for providing translational research informatics support.* J Biomed Inform, 2009. **42**(2): p. 377-81.
54. Davidson, J.R., et al., *Escitalopram in the treatment of generalized anxiety disorder: Double-blind, placebo controlled, flexible-dose study.* Depression and anxiety, 2004. **19**(4): p. 234-240.
55. Rothmann, M.D., B.L. Wiens, and I.S. Chan, *Design and analysis of non-inferiority trials*. 2012: Chapman & Hall/CRC.
56. Hardin, J.W., J.M. Hilbe, and J. Hilbe, *Generalized linear models and extensions*. 2007: Stata press.
57. Gadermann, A.M., et al., *Comorbidity and disease burden in the National Comorbidity Survey Replication (NCS-R).* Depress Anxiety, 2012. **29**(9): p. 797-806.
58. Shear MK, et al., Multicenter collaborative panic disorder severity scale. Am J Psychiatry, 1997. 154: p. 1571-75.
59. Shear MK, Vander Bilt J, Rucci P, Endicott J, Lydiard B, Otto MW, Pollack MH, Chandler L, Williams J, Ali A, Frank DM. Reliability and validity of a structured interview guide for the Hamilton Anxiety Rating Scale (SIGH-A). Depress Anxiety. 2001;13(4):166-78.

60. Heimberg, R. G., Horner, K. J., Juster, H. R., Safren, S. A., Brown, E. J., Schneier, F. R., & Liebowitz, M. R. (1999). Psychometric properties of the Liebowitz Social Anxiety Scale. Psychological Medicine, 29(1), 199-212.

61. Bernstein DP, Ahluvalia T, Pogge D, Handelsman L. Validity of the Childhood Trauma Questionnaire in an adolescent psychiatric population. J Am Acad Child Adolesc Psychiatry. 1997 Mar;36(3):340-8.

62 Gray MJ, Litz BT, Hsu JL, Lombardo TW. Psychometric properties of the life events checklist. Assessment. 2004 Dec;11(4):330-41.

63 Prigerson HG, Maciejewski PK, Reynolds CF 3rd, Bierhals AJ, Newsom JT, et al. Inventory of Complicated Grief: a scale to measure maladaptive symptoms of loss. Psychiatry Res. 1995 Nov 29;59(1-2):65-79.64 Carlson, L. E., M. Speca, K. D. Patel, and E. Goodey. 2003. 'Mindfulness-based stress reduction in relation to quality of life, mood, symptoms of stress, and immune parameters in breast and prostate cancer outpatients', *Psychosom Med*, 65: 571-81.

64 Neff, K. D. (2003). "Development and validation of a scale to measure self-compassion." Self and Identity 2: 223-250.
