# Supplementary material for: Mindfulness Meditation vs Escitalopram for Treatment of Anxiety Disorders: Secondary Analysis of a Randomized Clinical Trial
Source: JAMA Netw Open. 2024 Oct 9;7(10):e2438453. doi: 10.1001/jamanetworkopen.2024.38453 (PMC11581486; doi:10.1001/jamanetworkopen.2024.38453)
Supplement: Supplement 2. — eMethods eFigure. CONSORT Flow Diagram [file jamanetwopen-e2438453-s002.pdf]

Supplementary Online Content

Hu H, Mete M, Rustgi N, et al. Mindfulness meditation vs escitalopram for treatment of anxiety disorders: secondary analysis of a randomized clinical trial. *JAMA Netw Open*. 2024;7(10):e2438453. doi:10.1001/jamanetworkopen.2024.38453

eMethods

eReferences

eFigure. CONSORT Flow Diagram

This supplementary material has been provided by the authors to give readers additional information about their work.

© 2024 Hu H et al. *JAMA Network Open.*

eMethods

Participant Recruitment

Adults 18-75 years old were recruited from the community using IRB-approved study flyers, social media advertisments, and print and radio recruitment materials in Boston, New York, and Washington D.C. Diagnostic interviews were conducted by trained clinicians (M.D, PhD, MSW or Nurse Practitioner) using a structured psychiatric diagnostic interview [1]. Inclusion criteria included having a primary diagnosis of one of the main anxiety disorders (generalized anxiety disorder (GAD), social anxiety disorder (SAD), panic disorder, or agoraphobia). Comorbid depression was allowed as long as the anxiety disorder was considered primary. Exclusion criteria included presence of psychotic disorders, obsessive compulsive disorder, eating disorders, bipolar disorder, developmental or organic mentor disorders, current post-traumatic stress disorder or substance use disorder. Participants could not be taking antidepressants, mood stabilizers, barbiturates, or antipsychotic medications, with the exception of trazodone (if 100 mg or less) and only if it was being used for insomnia. Benzodiazepine use, however, was allowed, if the dose was stable over the previous 4 weeks (or more) prior to baseline and the patient planned to continue at the same dose through the study. In addition, patients who started psychotherapy within 1 month of the screen interview, or who were engaged in ongoing psychotherapy of any duration directed specifically toward the treatment of anxiety (such as cognitive-behavioral therapy for anxiety) were excluded.

© 2024 Hu H et al. *JAMA Network Open.*

Assessments

Study participants were seen by study clinicians at the research clinic for each of the three sites (Georgetown University Medical Center, in Washington DC, New York University Langone in New York City, NY, and Massachusetts General Hospital in Boston, MA). Blinded clinical evaluators conducted the clinical ratings for the Structured Interview Guide for the Hamilton Anxiety Scale (SIGH-A) [2], the Panic Disorder Severity Scale (PDSS) [3], and the Liebowitz Social Anxiety Scale (LSAS) [4]. Patient-reported anxiety symptom measures outcomes were the Beck Anxiety Inventory (BAI) [5], the PROMIS Anxiety Short Form [6], and the Penn State Worry Questionnaire [7].

eReferences

1. Hoge EA, Bui E, Mete M, et al. Treatment for anxiety: Mindfulness meditation versus escitalopram (TAME): Design of a randomized, controlled non-inferiority trial. Contemp Clin Trials. 2020;91:105965. doi:10.1016/j.cct.2020.105965.

2. Shear MK, Vander Bilt J, Rucci P, et al. Reliability and validity of a structured interview guide for the Hamilton Anxiety Rating Scale (SIGH-A). Depress Anxiety. 2001;13(4):166-178.

3. Shear MK, Brown TA, Barlow DH, et al. Multicenter collaborative panic disorder severity scale. Am J Psychiatry. 1997;154(11):1571-1575. doi:10.1176/ajp.154.11.1571

4. Heimberg RG, Horner KJ, Juster HR, et al. Psychometric properties of the Liebowitz Social Anxiety Scale. Psychol Med. 1999;29(1):199-212. doi:10.1017/s0033291798007879

5. Beck AT, Epstein N, Brown G, Steer RA. An inventory for measuring clinical anxiety: psychometric properties. J Consult Clin Psychol. 1988;56(6):893-897. doi:10.1037//0022-006x.56.6.893

© 2024 Hu H et al. *JAMA Network Open.*

6. Pilkonis PA, Choi SW, Reise SP, et al. Item banks for measuring emotional distress from the Patient-Reported Outcomes Measurement Information System (PROMIS®): depression, anxiety, and anger. Assessment. 2011;18(3):263-283. doi:10.1177/1073191111411667

7. Meyer TJ, Miller ML, Metzger RL, Borkovec TD. Development and validation of the Penn State Worry Questionnaire. Behav Res Ther. 1990;28(6):487-495. doi:10.1016/0005-7967(90)90135-6

© 2024 Hu H et al. *JAMA Network Open.*

eFigure. CONSORT Flow Diagram

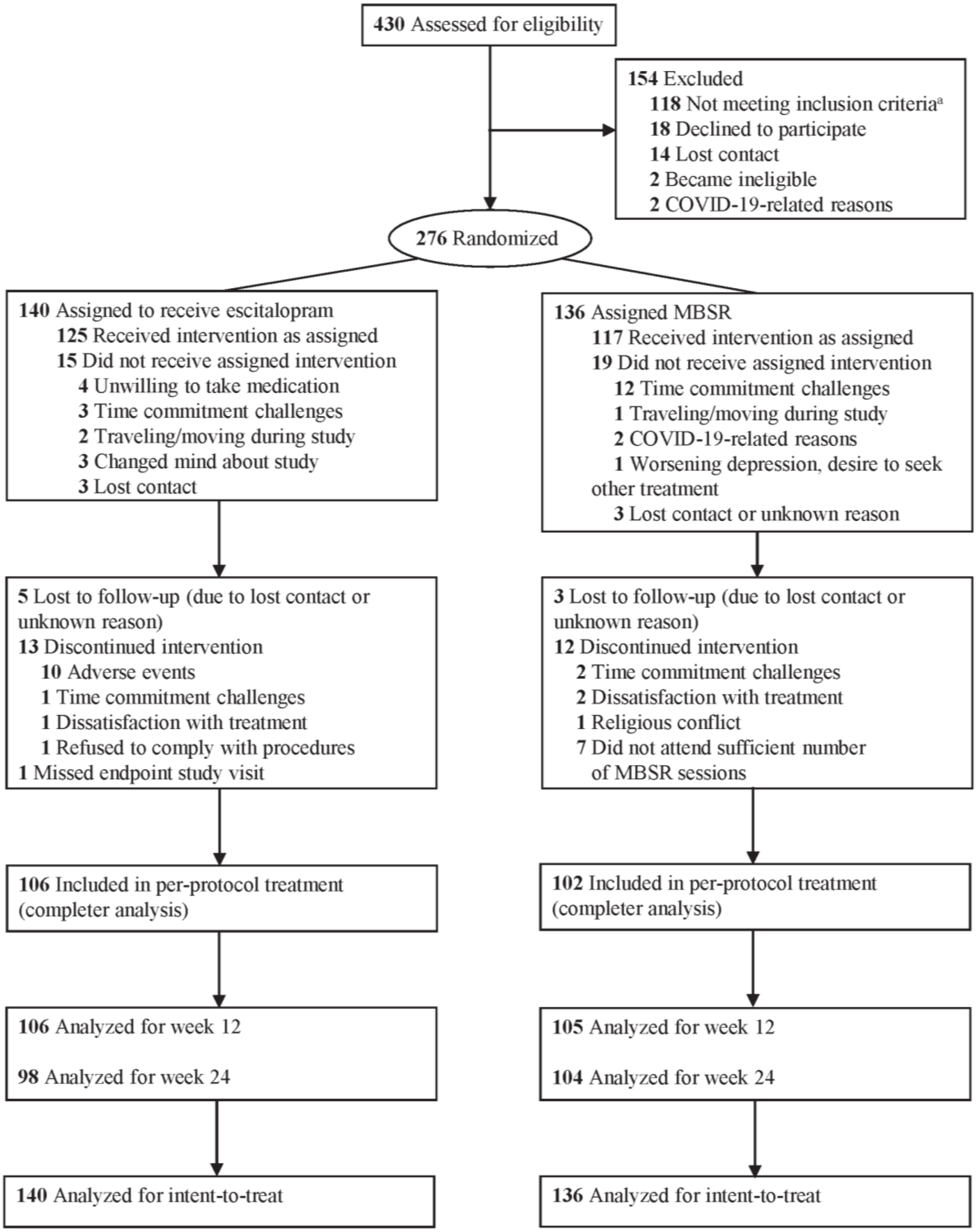

Figure 1: CONSORT diagram

[a]Reasons for not meeting inclusion criteria: having an excluded psychiatric diagnosis (n=49); no anxiety disorder (n=35); medi conditions (n=12); not willing to follow, or unable to understand study procedures (n=11); high risk for suicidality (n=4); currently taking disallowed medication (n=3); recently initiated psychotherapy (n=3); and already completed MBSR training or equivalent (n=1)

© 2024 Hu H et al. *JAMA Network Open.*
