# Supplementary material for: Mindfulness Meditation vs Escitalopram for Treatment of Anxiety Disorders: Secondary Analysis of a Randomized Clinical Trial
Source: JAMA Netw Open. 2024 Oct 9;7(10):e2438453. doi: 10.1001/jamanetworkopen.2024.38453 (PMC11581486; doi:10.1001/jamanetworkopen.2024.38453)
Supplement: Supplement 3. — Data Sharing Statement [file jamanetwopen-e2438453-s003.pdf]

Data Sharing Statement

Hu. Mindfulness Meditation vs Escitalopram for Treatment of Anxiety Disorders. *JAMA Netw Open*. Published October 09, 2024. doi:10.1001/jamanetworkopen.2024.38453

Data
Data available: No

Additional Information
Explanation for why data not available: Unfortunately at the time the data were collected, the IRB approved consent form did not allow for release of individual patient data.
